# Pan-Cancer Analysis of PIMREG as a Biomarker for the Prognostic and Immunological Role

**DOI:** 10.3389/fgene.2021.687778

**Published:** 2021-09-14

**Authors:** Hua Zhu, Xinyao Hu, Yingze Ye, Zhihong Jian, Yi Zhong, Lijuan Gu, Xiaoxing Xiong

**Affiliations:** ^1^Department of Neurosurgery, Renmin Hospital of Wuhan University, Wuhan, China; ^2^Central Laboratory, Renmin Hospital of Wuhan University, Wuhan, China; ^3^Department of Oncology, Renmin Hospital of Wuhan University, Wuhan, China; ^4^Department of Anesthesiology, Renmin Hospital of Wuhan University, Wuhan, China

**Keywords:** pan-cancer, prognosis, TMB, MSI, tumor immunity, PIMREG

## Abstract

Phosphatidylinositol binding clathrin assembly protein interacting mitotic regulator (PIMREG) localizes to the nucleus and can significantly elevate the nuclear localization of clathrin assembly lymphomedullary leukocythemia gene. Although there is some evidence to support an important action for PIMREG in the occurrence and development of certain cancers, currently no pan-cancer analysis of PIMREG is available. Therefore, we intended to estimate the prognostic predictive value of PIMREG and to explore its potential immune function in 33 cancer types. By using a series of bioinformatics approaches, we extracted and analyzed datasets from Oncomine, The Cancer Genome Atlas, Cancer Cell Lineage Encyclopedia (CCLE) and the Human Protein Atlas (HPA), to explore the underlying carcinogenesis of PIMREG, including relevance of PIMREG to prognosis, microsatellite instability (MSI), tumor mutation burden (TMB), tumor microenvironment (TME) and infiltration of immune cells in various types of cancer. Our findings indicate that PIMREG is highly expressed in at least 24 types of cancer, and is negatively correlated with prognosis in major cancer types. In addition, PIMREG expression was correlated with TMB in 24 cancers and with MSI in 10 cancers. We revealed that PIMREG is co-expressed with genes encoding major histocompatibility complex, immune activation, immune suppression, chemokine and chemokine receptors. We also found that the different roles of PIMREG in the infiltration of different immune cell types in different tumors. PIMREG can potentially influence the etiology or pathogenesis of cancer by acting on immune-related pathways, chemokine signaling pathway, regulation of autophagy, RIG-I like receptor signaling pathway, antigen processing and presentation, FC epsilon RI pathway, complement and coagulation cascades, T cell receptor pathway, NK cell mediated cytotoxicity and other immune-related pathways. Our study suggests that PIMREG can be applied as a prognostic marker in a variety of malignancies because of its role in tumorigenesis and immune infiltration.

## Introduction

Malignant cancer is a major cause of death and a leading stumbling block to patients’ living quality in most countries worldwide, but to date, there is no absolute cure for malignant tumor (Bray et al., 2018). Recently, tumor immunotherapy has emerged as a new approach to tumor treatment, particularly immune checkpoint blockade therapy (Ribas and Wolchok, 2018). The emergence and refinement of gene expression databases has made it promising to explore novel immunotherapeutic targets by pan-cancer expression analysis of particular genes and assessing their relevance to patients’ clinical prognosis and associated pathological mechanisms (Blum et al., 2018).

Phosphatidylinositol binding clathrin assembly protein interacting mitotic regulator (PIMREG), also referred to as FAM64A, RCS1, and CATS, was first identified in 2006 during a screen for proteins that interplay with lymphoid myeloid clathrin assembly protein (Archangelo et al., 2006). In normal tissues, a variety of northern blots demonstrated that PIMREG was primarily expressed in the thymus, colon and spleen (Archangelo et al., 2006). PIMREG has been shown to control the transition from the metaphase to anaphase in the cell division and can be regarded as a marker for multiplication (Archangelo et al., 2008; Zhao et al., 2008; Barbutti et al., 2016), showing a role in the development of cancer cell (Jiang Z.M. et al., 2020). Moreover, a previous study claimed that PIMREG expressed highly protein levels in cancer cells such as lymphoma and leukemia, but hardly expressed in lymphocytes from peripheral blood or non-proliferative T cells (Archangelo et al., 2008). Moreover, PIMREG was verified to promote breast cancer (BRCA) aggressiveness through activation of the NF-κB pathway, suggesting that it may be a novel prognostic indicator for BBCA (Jiang et al., 2019; Sun et al., 2019). A previous study identified PIMREG as a biomarker of proliferation that facilitated aggressive development of bile duct cancer (CHOL) in part by regulation of cell cycle-related biomarkers (Jiang Z.M. et al., 2020). Additionally, high PIMREG expression may be regarded as a risk element for prognostic deterioration of pancreatic cancer (PAAD) (Jiao et al., 2019). It has also been reported that PIMREG is related to the survival in the clear cell renal cell carcinoma (Wei et al., 2019) and prostate cancer (Zhou et al., 2021). However, the prognostic predictive value of PIMREG remains unstudied in some cancer types. More works are urgently required to explore the role of PIMREG in various cancers.

There is also a complicated interface between malignant cancers and their microenvironment. Infiltrated immune cells are known to be important components of the TME which comprises innocent and adaptive immune cells, consisting of natural killer (NK) cells, neutrophils, macrophages and dendritic cells (DC), etc. Tumor cells are subject to the surveillance of immune cells throughout their life, and cancer develops and progresses only when the immune cells failure to destroy preneoplastic cells (Carlsten and Järås, 2019). Presently, various effective chemotherapy and radiotherapy are used to restore immune surveillance by the activation of the immune response (Zitvogel et al., 2013). With the development and refinement of immunotherapies, promising targets are gradually being discovered. For example, studies have shown that zinc finger homeobox three mutations is verified as an independent predictive biomarker for non-small cell pulmonary cancer and can be applied as novel predictive marker in guiding immune checkpoint inhibitor treatment of non-small cell lung cancer (Zhang et al., 2021). Additionally, angiopoietin-2 may be used as a therapeutic target of immune checkpoint treatment in patients with advanced cancer (Leong and Kim, 2020; Wu X. et al., 2017). A previous study has suggested that PIMREG regulates Th17 differentiation and colitis and inflammation-associated cancer by modulating transcriptional activity of STAT3, indicating that PIMREG may be correlated with immune response in the tumorigenesis (Xu C. et al., 2020). In addition, PIMREG is primarily expressed in the thymus and spleen (Archangelo et al., 2006) which play crucial roles in immune system. Therefore, we speculate that PIMREG may play a role in cancer development by regulating immune system-related functions. However, poor response to immunotherapy can lead to a poor prognosis. Therefore, further discovery of more specific or universal immune targets for cancer immunotherapies is still required.

Our study extracted datasets from several databases such as HPA, Oncomine, TGCA and CCLE to investigated the PIMREG expression levels and its correlation with prognosis and immune response in various cancers. Our findings suggested that PIMREG may affect the prognosis of patients with certain cancer types, partially through its interplay with the infiltration of immune cells.

## Materials and Methods

### Data Processing and Differential Expression Analysis

Oncomine^1^, an online cancer microarray database has approximately 48 million gene expression measures and over 80,000 samples of different cancer types (Rhodes et al., 2004). We used this database to analyze the mRNA expression of PIMREG in 33 types of human malignances. Filters were set as: gene symbol, “PIMREG,” datatype “mRNA,” and cancer vs. normal analysis. Thresholds included: gene rank: 10%, fold change: 1.5, and *p*-value: 0.001. Data sets with statistical significances were noted.

We downloaded 33 cancer-related RNA sequences, clinicopathological and survival data on UCSC Xena website^2^. We then extracted and integrated PIMREG expression data in TCGA^3^by Perl software and performed pan-cancer analysis. The ‘‘wilcox.test’’ method was applied to investigate the different mRNA expression levels of PIMREG in pan-cancer. Thereafter, we investigated the mRNA sequencing in different cancer cell lines from Cancer Cell Line Encyclopedia (CCLE^4^). The cut-off was set as a False Discovery Rate (FDR) value < 0.05. The R package “ggpubr” was applied to design the box diagram.

### Immunohistochemistry Staining

Immunohistochemical images of PIMREG protein expression analyses, assessment of the differences in PIMREG expression at the protein level, were performed in normal and ten tumor tissues, including liver cancer (LIHC), bladder cancer (BLCA), lung adenocarcinoma (LUAD), lung squamous cell carcinoma (LUSC), glioblastoma (GBM), ovarian cancer (OV), PAAD, stomach cancer (STAD), testicular cancer (TGCT), endometrioid cancer (UCEC) from the HPA^5^. The anti-body used for IHC was HPA043783. The number with IHC of the tumor samples was 10–12. In the HPA dataset, antibody staining in the cancer types in the current human tissue is reported as not detected, low, medium, or high. This score is based on the staining intensity and fraction of stained cells.

### Identification of the Correlations Between PIMREG Expression Levels and Clinicopathology or Survival in Human Cancers

We extracted the survival information for each sample in the TCGA. We then selected several indicators: overall survival (OS), disease-specific survival (DSS), disease-free interval (DFI), and progression-free interval (PFI), to clarify the association of PIMREG expression with the prognosis of patients with various cancers. We used the Kaplan–Meier (KM) method and log-rank test for survival analysis of 33 cancer types (*p* < 0.05) and then plotted survival curves using R packages “survminer” and “survival.” Subsequently, R packages “survival” and “forestplot” were used for Cox analysis to identification the correlation of PIMREG with survival. The R packages “ggpubr” and “limma” were used for clinicopathological correlation analysis. The cut off was median expression to define the high or low expression.

### Association Between PIMREG Expression and Tumor Mutation Burden or Microsatellite Instability Across Cancers

To calculate the number of mutations in 33 cancers from somatic mutation datasets. TMB was evaluated based on Perl scripts and divided by the exon length for correction. The MSI scores were extracted using TCGA. Relationship of PIMREG expression with TMB or MSI was analyzed using the “cor.test” command based on the Spearman’s method. The two metrics are visualized by radar plots, which were devised by applying the R package “fmsb.”

### Association Between PIMREG Expression and Tumor Immune Microenvironment or Infiltration of Immune Cells in Tumors

Subsequently, we applied the ESTIMATE algorithm in the R package “estimate” and “limma” to calculate immune and stromal scores (Diboun et al., 2006). We analyzed tumor purity and the infiltration of stromal/immune cells in the tissue of various tumors (*n* = 33) based on PIMREG expression data using CIBERSORT, which was developed to estimate the abundance of particular cells in hybrid cell populations applying gene expression datasets (Newman et al., 2015). We next analyzed the correlation of PIMREG with TME or infiltration of immune cells by using R packages “ggplot2,” “ggpubr,” and “ggExtra” (with a cut-off value of *p* < 0.001).

### Co-expression of PIMREG With Immune-Related Genes and Pathways in Tumors

R packages ‘‘limma,’’ ‘‘reshape2,’’ and ‘‘RColorBrewer’’ were applied to perform the co-expression analysis. Gene ontology (GO) and Kyoto Encyclopedia of Genes and Genomes (KEGG) gene sets were obtained on the Gene Set Enrichment Analysis web (GSEA,^6^). Subsequently, the GO and KEGG functional annotations of PIMREG and the enrichment pathway were analyzed using R package “limma,” “org.Hs.eg.db,” “clusterProfiler” (Yu et al., 2012) and “enrichplot.”

### Statistical Analysis

All gene expression data were subjected to log2 transformative normalization. The comparison of normal tissues and cancerous tissues was performed by two-group *t*-test. The KM analyses, Cox proportional hazards model and log-rank test were conducted for all survival analyses in our work. Correlations between two variables were analyzed using Spearman’s test or Pearson’s test; *p* < 0.05 was defined as a significant difference. All the statistical analyses were conducted by R software (version 4.0.2).

## Results

### Different Expression Levels Between Normal and Tumors Tissues

**FIGURE 1:**
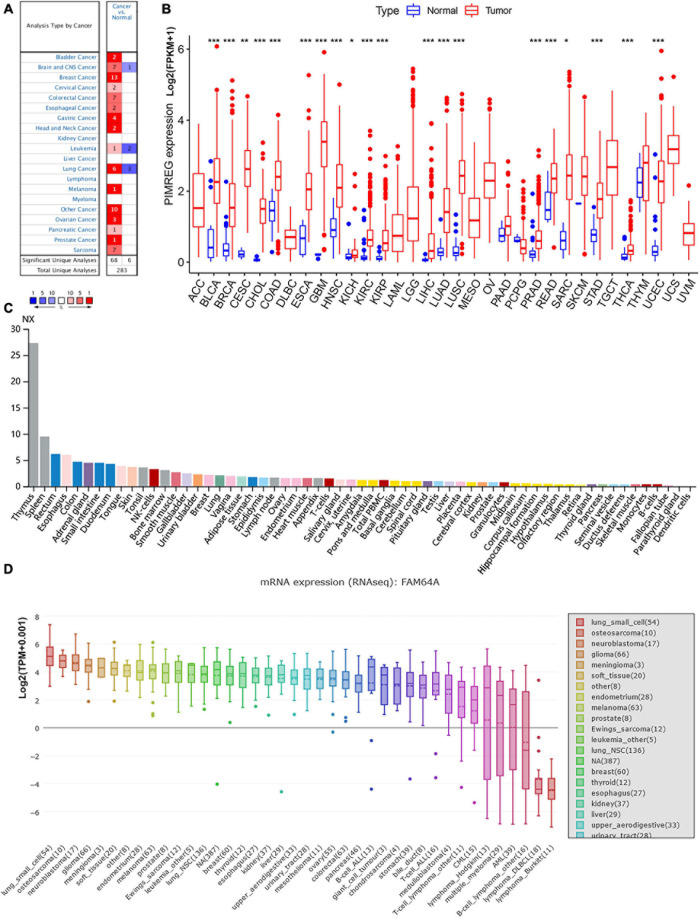
Different expression between normal and tumors tissues. **(A)** PIMREG gene expression is increased in major tumor tissues compared to normal tissues. The number in each small rectangle represents the number of data sets with high or low expression of the PIMREG gene in each cancer. The red and blue shades indicate the proportion of data sets with high or low expression of PIMREG in each cancer tissue, respectively. The numbers 68 and 6 in the small white box represent the total number of data sets with high and low expression of PIMREG gene in cancer tissues, and 283 represents the total number of all data sets included. **(B)** Different PIMREG expression levels in various tumor and normal tissues. **(C)** Different protein levels of PIMREG in various organs. Consensus Normalized expression (NX) levels for 55 tissue types and 6 blood cell types, created by combining the data from the three transcriptomics datasets (HPA, GTEx and FANTOM5) using the internal normalization pipeline. **(D)** mRNA expression of PIMERG in different cell lines. *p < 0.05, **p < 0.01, and ***p < 0.001.

We used Oncomine to investigate PIMREG expression levels in normal and various cancer tissues. We discovered that PIMREG expression was markedly increased in most cancer types, including bladder, brain and central nervous system (CNS), breast, cervical, colorectal, esophageal, gastric, head and neck, leukemia, lung, melanoma, ovarian prostate, pancreatic, prostate, sarcoma and other cancers. Interestingly, lower expression of PIMREG was also observed in cancer datasets, including brain and CNS, leukemia, and lung cancers (Figure 1A). The paradoxical results were attributed to different data collection methods and putative mechanisms with different biological properties.

To further evaluate the expression of PIMREG in pan-cancer, RNA sequencing data obtained from the TCGA were analyzed using R software. A total of 11,057 TCGA profiles (included 730 normal and 10,327 tumor samples) of mRNA expression for 33 cancers were gained. Supplementary Table 1 showed the amount of different cancer and normal samples contained in this study, and Supplementary Table 2 showed the expression profiles of PIMREG in 33 cancer categories. Our findings showed that PIMREG was expressed higher in 20 cancers, including BLCA, BRCA, cervical cancer (CESC), bile duct cancer (CHOL), colon cancer (COAD), esophageal cancer (ESCA), GBM, head and neck cancer (HNSC), kidney chromophobe (KICH), kidney clear cell carcinoma (KIRC), kidney papillary cell carcinoma (KIRP), LIHC, LUAD, LUSC, prostate cancer (PRAD), rectal cancer (READ), sarcoma (SARC, STAD), thyroid cancer (THCA), and (endometrioid cancer) UCES. Meanwhile, a lower PIMREG expression was not found in any type of the 33 cancers compared with the normal tissues. But no significant difference in PIMREG expression was observed between PAAD and normal tissues, thymoma (THYM) and normal tissues. No significant difference in certain cancers with only few normal samples (e.g., only two normal tissue samples in TGCT), probably due to the small sample size (Figure 1B). However, we found that the expression levels of PIMREG were high in TGCT cancers. Hence, it is required to investigate the expression of PIMREG in normal tissues in other databases. We further investigated the PIMREG expression in normal tissues according to the HPA database. We found that in normal tissues, the expression of PIMREG is highest in thymus compared with other normal organs (Figure 1C). Meanwhile, we further investigated the mRNA sequence of PIMREG gene in 33 types of cancers in CCLE. The four cancer cell lines with the highest PIMREG mRNA expression levels were lung small cell, osteosarcoma, neuroblastoma, and glioma (Figure 1D), which confirmed our assessment of RNA sequencing data in the TCGA database.

**FIGURE 2:**
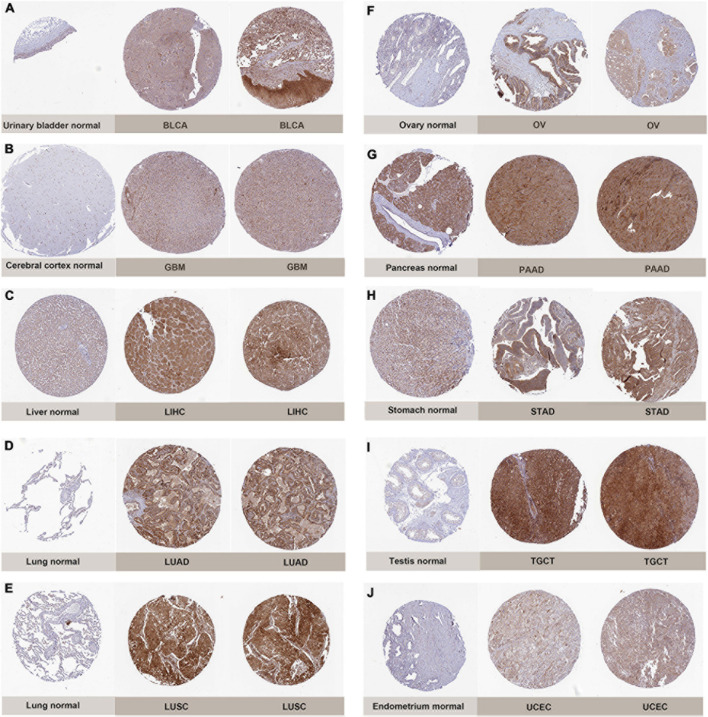
Representative immunohistochemical stainings in various normal (left) and tumor (right) tissues. The protein expression of PIMREG was markedly higher in bladder Cancer (BLCA), glioblastoma (GBM), liver Cancer (LIHC), lung adenocarcinoma (LUAD), lung squamous cell carcinoma (LUSC), ovarian cancer (OV), pancreatic cancer (PAAD), stomach cancer (STAD), testicular cancer (TGCT) and endometrioid cancer (UCEC). **(A)** Urinary bladder. **(B)** Cerebral cortex. **(C)** Liver. **(D,E)** Lung. **(F)** Ovary. **(G)** Pancreas. **(H)** Stomach. **(I)** Testis. **(J)** Endometrium.

Subsequently, to determine the expression of PIMREG at protein level, the IHC results from the HPA were analyzed and compared with the PIMREG gene expression datasets provided by TCGA. Figures 2A–J, the data analysis results from the two databases were consistent with each other, and the HPA database complemented the deficiency of the data of PIMREG expression in some normal tissues in TCGA. That is, the TGCA database didn’t have the peri-carcinomatous tissue sample of OV and TGCT, which was contained in the HPA database. Normal urinary bladder, cerebral cortex, ovary and endometrium had weak PIMREG IHC staining, while BLCA, GBM, OV, and UCES tissues had medium PIMREG IHC staining. The PIMREG IHC staining was weak in normal lung, testis, while LUAD, LUSC, and TGCT tissues had strong staining. Although liver, pancreas and stomach had medium PIMREG IHC staining, the PIMREG IHC staining was strong in LIHC, PAAD, and STAD.

### Prognostic Value of PIMREG in Pan-Cancers

**FIGURE 3:**
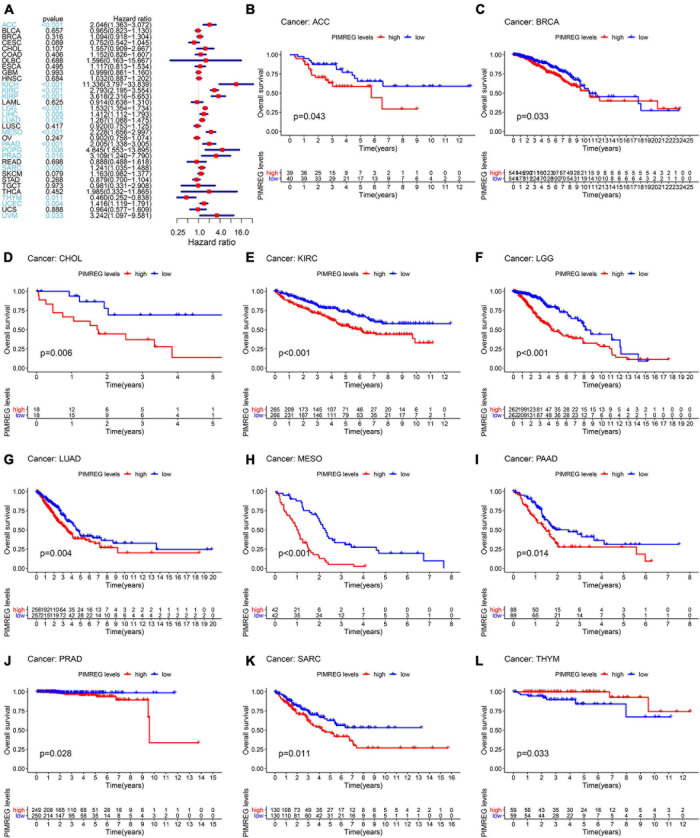
Correlation of PIMREG expression with overall survival time (OS). **(A)** Forest plots of OS correlations in 33 cancer categories. **(B–L)** KM analyses of the correlation of PIMREG expression with OS.

To further explore the correlation of PIMREG expression with prognosis, survival association analyses, including OS, DSS, DFI, and PFI were performed in 33 cancers. Cox analysis indicated that the expression levels of PIMREG had closely correlations with OS in adrenocortical cancer (ACC) (*p* < 0.001), KICH (*p* < 0.001), KIRC (*p* < 0.001), KIRP (*p* < 0.001), LGG (*p* < 0.001), LIHC (*p* = 0.005), LUAD (*p* = 0.002), mesothelioma (MESO) (*p* < 0.001), PAAD (*p* < 0.001), pheochromocytoma and paraganglioma (PCPG) (*p* = 0.006), PRAD (*p* = 0.016), SARC (*p* = 0.020), THYM (*p* = 0.011), UCEC (*p* = 0.004), ocular melanomas (UVM) (*p* = 0.033) (Figure 3A). Moreover, PIMREG was a high-risk gene in ACC, KICH, KIRC, KIRP, LGG, LUAD, LIHC, LUAD, MESO, PAAD, PCPG, PRAD, SARC, UCEC, and UVM, particularly KICH (hazard ratio = 11.336), while it was a gene of low risk in THYM. In addition, KM plotter results showed that among the individuals with ACC (Figure 3B, *p* = 0.043), BRCA (Figure 3C, *p* = 0.033), CHOL (Figure 3D, *p* = 0.006), KIRC (Figure 3E, *p* < 0.001), LGG (Figure 3F, *p* < 0.001), LUAD (Figure 3G, *p* = 0.004), MESO (Figure 3H, *p* < 0.001), PAAD (Figure 3I, *p* = 0.014), PRAD (Figure 3J, *p* = 0.028), and SARC (Figure 3K, *p* = 0.011), those with high PIMREG expression had less time of survival, while the individuals with TYHM (Figure 3L, *p* = 0.033) had a longer survival time.

**FIGURE 4:**
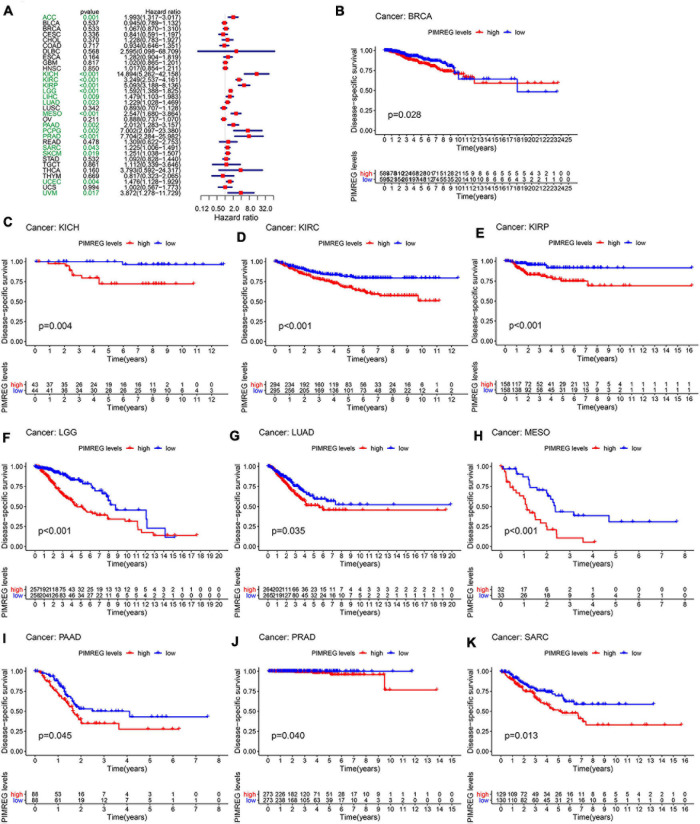
Correlation between the expression of PIMREG and disease-specific survival (DSS). **(A)** Forest plots of PIMREG expression in 33 tumors in association with DSS. **(B–K)** KM analyses of the relationship between the expression of PIMREG and DSS.

**FIGURE 5:**
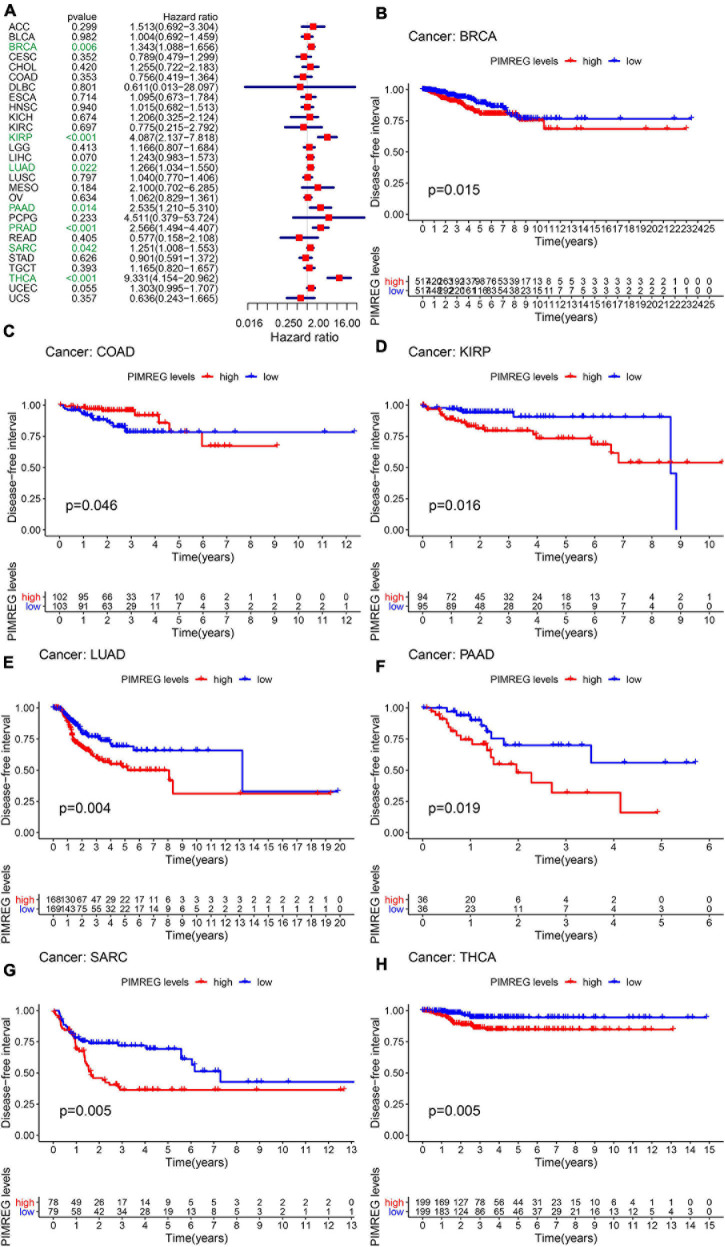
Correlation between the expression of PIMREG and disease-free interval (DFI). **(A)** Forest plots of the expression of PIMREG in 33 tumors in association with DFI. **(B–H)** KM analyses of the relationship between PIMREG expression and DFI.

**FIGURE 6:**
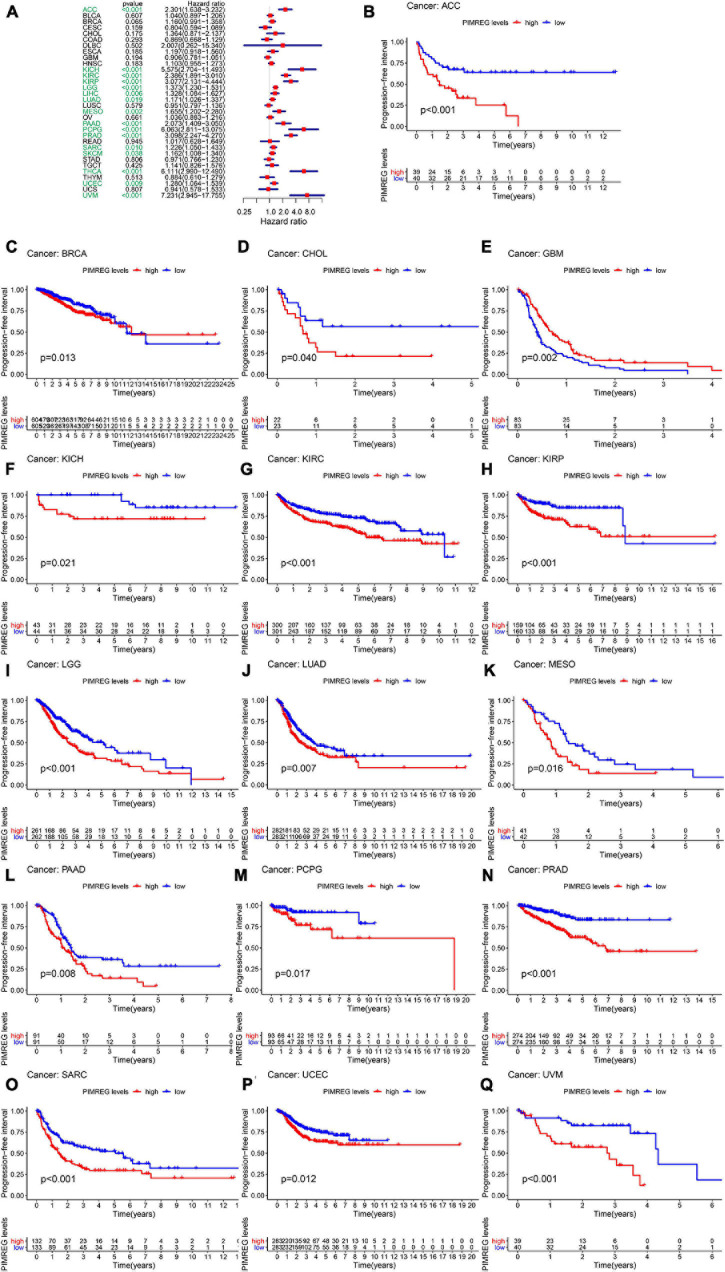
Correlation between the expression of PIMREG and progression-free interval (PFI). **(A)** Forest plots of PIMREG expression levels in 33 tumors in association with PFI. **(B–Q)** KM analyses of the relationship of PIMREG expression with PFI.

Furthermore, DSS analyses (Figure 4A) showed a correlation between high PIMREG expression and adverse outcomes in the patients with ACC (*p* = 0.001), KICH (*p* < 0.001), KIRC (*p* < 0.001), KIRP (*p* < 0.001), LGG (*p* < 0.001), LIHC (*p* = 0.009), LUAD (*p* = 0.023), MESO (*p* < 0.001), PAAD (*p* = 0.002), PCPG (*p* = 0.002), PRAD (*p* < 0.001), SARC (*p* = 0.043), SKCM (*p* = 0.019), UCES (*p* = 0.004), and UVM (*p* = 0.017). KM analyses also demonstrated a correlation of high PIMREG expression levels with adverse prognosis in patients with BRAC (Figure 4B, *p* = 0.028), KICH (Figure 4C, *p* = 0.004), KIRC (Figure 4D, *p* < 0.001), KIRP (Figure 4E, *p* < 0.001), LGG (Figure 4F, *p* < 0.001), LUAD (Figure 4G, *p* = 0.035), MESO (Figure 4H, *p* < 0.001), PAAD (Figure 4I, *p* = 0.045), PRAD (Figure 4J, *p* = 0.040), and SARC (Figure 4K, *p* = 0.013). Correlation between high PIMREG expression and poor DFI was detected in BRCA (*p* = 0.006), KIRP (*p* < 0.001), LUAD (*p* = 0.022), PAAD (*p* = 0.014), PRAD (*p* < 0.001), SARC (*p* = 0.042), THCA (*p* < 0.001) (Figure 5A). Furthermore, KM survival analysis revealed that the significant relationships in BRCA (Figure 5B, *p* = 0.015), COAD (Figure 5C, *p* = 0.048), KIRP (Figure 5D, *p* = 0.016), LUAD (Figure 5E, *p* = 0.004), PAAD (Figure 5F, *p* = 0.019), SARC (Figure 5G, *p* = 0.005), and THCA (Figure 5H, *p* = 0.005). The forest plots revealed the correlation of high PIMREG expression with poor PFI in ACC (*p* < 0.001), KICH (*p* < 0.001), KIRC (*p* < 0.001), KIRP (*p* < 0.001), LGG (*p* < 0.001), LIHC (*p* = 0.006), LUAD (*p* = 0.019), MESO (*p* = 0.002), PAAD (*p* < 0.001), PCPG (*p* < 0.001), PRAD (*p* < 0.001), SARC (*p* = 0.010), SKCM (*p* = 0.038), THCA (*p* < 0.001), UCEC (*p* = 0.009), and UVM (*p* < 0.001) (Figure 6A). KM analyses indicated that patients with ACC (Figure 6B, *p* < 0.001), BRCA (Figure 6C, *p* = 0.013), CHOL (Figure 6D, *p* = 0.040), KICH (Figure 6F, *p* < 0.021), KIRC (Figure 6G, *p* < 0.001), KIRP (Figure 6H, *p* < 0.001), LGG (Figure 6I, *p* < 0.001), LUAD (Figure 6J, *p* = 0.007), MESO (Figure 6K, *p* < 0.016), PAAD (Figure 6L, *p* = 0.008), PCPG (Figure 6M, *p* = 0.017), and PRAD (Figure 6N, *p* < 0.001), SARC (Figure 6O, *p* < 0.001), UCEC (Figure 6P, *p* = 0.012), and UVM (Figure 6Q, *p* < 0.001) and low levels expression of PIMREG had longer time of survival, while individuals with GBM (Figure 6E, *p* = 0.002) and low PIMREG expression had poor PFI.

### Correlation Between PIMREG Expression and Pan-Cancer Clinicopathology

**FIGURE 7:**
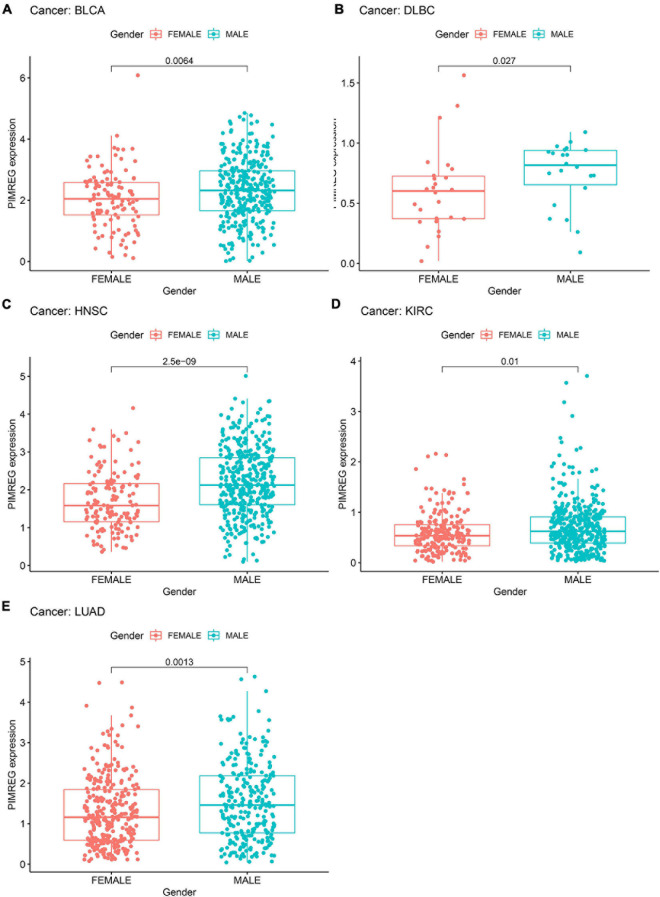
Association of PIMREG expression with gender in panel **(A)** bladder cancer (BLCA), **(B)** large B-cell lymphoma (DLBC), **(C)** head and neck cancer (HNSC), **(D)** kidney clear cell carcinoma (KIRC), and **(E)** lung adenocarcinoma (LUAD).

**FIGURE 8:**
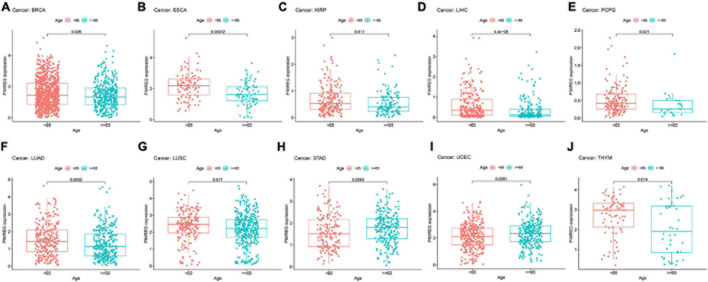
Relationship between the expression of PIMERG and age in panel **(A)** breast cancer (BRCA), **(B)** esophageal cancer (ESCA), **(C)** kidney papillary cell carcinoma (KIRP), **(D)** liver cancer (LIHC), **(E)** pheochromocytoma and paraganglioma (PCPG), **(F)** lung adenocarcinoma (LUAD), **(G)** lung squamous cell carcinoma (LUSC), **(H)** stomach cancer (STAD), **(I)** endometrioid cancer (UCEC), and **(J)** thymoma (THYM).

We next investigated the differences in PIMREG expression between the male and female patients with 27 types cancers (except BRCA, CESC, UCEC, OV, PRAD, TGCT, and UCS) (Figure 7 and Supplementary Figure 1). We found that the different expression levels of PIMREG only occurred in patients with 5 types cancers, including BLCA, HNSC, KIRC, DLBC, LUAD (Figure 7). Moreover, the expression levels of PIMREG in male patients with BLCA (Figure 7A, *p* = 0.0064), DLBC (Figure 7B, *p* = 0.027), HNSC (Figure 7C, *p* < 0.0001), KIRC (Figure 7D, *p* = 0.01) and LUAD (Figure 8E, *p* = 0.0013) were higher than the females. However, no significant differences in PIMERG expression were observed between genders in patients with other cancers.

Subsequently, we investigated the different expression levels of PIMREG based on age in patients with various types of tumors and revealed that patients aged ≥ 65 years with BRCA (Figure 8A, *p* = 0.026), ESCA (Figure 8B, *p* = 0.00012),KIRP (Figure 8C, *p* = 0.011), LIHC (Figure 8C, *p* < 0.001), PCPG (Figure 8D, *p* = 0.021, LUAD (Figure 8F, *p* = 0.0032), LUSC (Figure 8G, *p* = 0.017),THYM (Figure 8J, *p* = 0.014) had lower expression of PIMREG, while patients with STAD (Figure 8H, *p* = 0.0092) and UCEC (Figure 8I, *p* = 0.0091) ≥ 65 years had higher expression of PIMREG compared with patients < 65 years. No obvious correlation was found in other cancer patients between age and PIMERG expression (Supplementary Figure 2).

**FIGURE 9:**
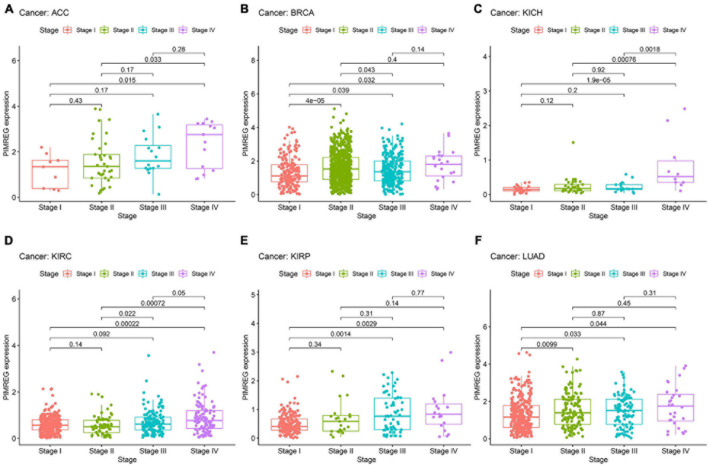
Association between the expression of PIMREG and tumor stage in panel **(A)** adrenocortical cancer (ACC), **(B)** breast cancer (BRCA), **(C)** kidney chromophobe (KICH), **(D)** kidney clear cell carcinoma (KIRC), **(E)** kidney papillary cell carcinoma (KIRP), and **(F)** lung adenocarcinoma (LUAD).

The correlation between tumor stage and PIMREG expression was analyzed, and PIMREG expression was found to be remarkably related to tumor stage in 12 cancers, including ACC, BRCA, COAD, ESCA, HNSC, KICH, KIRC, KIRP, LIHC, LUAD, TGCT, and THCA (Supplementary Figure 3). Of note, significant differences in PIMREG expression existed mainly between stage I and stage IV cancers (Figure 9). Intriguingly, in patients with ACC (Figure 9A, *p* = 0.015), BRCA (Figure 9B, *p* = 0.032), KICH (Figure 8C, *p* < 0.001), KIRC (Figure 8D, *p* < 0.001), KIRP (Figure 8E, *p* = 0.0029), and LUAD (Figure 9F, *p* < 0.044), the PIMREG expression were significantly increased stage IV compared with stage I. In addition, the PIMREG expression in stage III was also higher than stage I in BRCA (Figure 9B, *p* = 0.039), KIRP (Figure 9E, *p* = 0.0014), LUAD (Figure 9F, *p* = 0.033), LIHC (Supplementary Figure 3, *p* = 0.0035) and TGCT (Supplementary Figure 3, *p* < 0.001). Hence, we hypothesized that it was the high expression of PIMREG in these patients with advanced cancer that leaded to a lower survival time. Though the differences between stage I and IV were remarkable, the differences between other stage tumors were comparatively small (Figure 9 and Supplementary Figure 3) and no statistical significance was found in other cancers (Supplementary Figure 4).

### Association Between PIMREG Expression and TMB or MSI in Various Cancers

**FIGURE 10:**
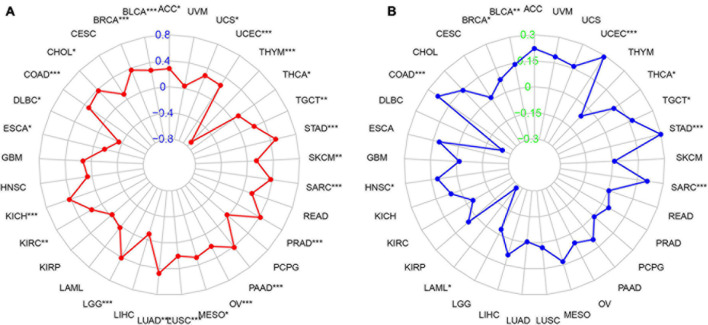
Association between PIMREG expression and the tumor mutational burden (TMB) and microsatellite instability (MSI) in various cancer types. **(A)** Radar plot showing the relationship of PIMREG expression with TMB in pan-cancers. Red lines represent correlation coefficients and blue values represent ranges. **(B)** Radar plot showing the relationship of PIMREG expression with MSI in pan-cancers. The blue lines represent correlation coefficients and green values represent ranges. *p < 0.05, **p < 0.01, and ***p < 0.001.

We then studied the associations between PIMREG expression and TMB and MSI, both of which are critically linked to the sensitive nature of the immune checkpoint inhibitors (ICI). Therefore, it is necessary to investigate the relationships between TMB and the expression of PIMREG in pan-cancers. The results showed that PIMREG expression related to TMB in a number of cancers (*n* = 24, *p* < 0.05). In particular, the expression of PIMREG positively corresponded to TMB in 22 cancer categories, including ACC, BLCA, BRCA, CHOL, COAD, KICH, KIRC, LGG, LUAD, LUSC, MESO, OV, PAAD, PRAD, SARC, SKCM, STAD, TGCT, THCA, UCEC, and UCS, while negatively correlated with TMB in DLBC, ESCA and THYM (Table 1 and Figure 10A). We further found that the expression of PIMREG was positively related to the MSI of 9 cancers, including BLCA, BRCA, COAD, HNSC, SARC, STAD, TGCT, THCA and UCEC, while had a negative correlation with MSI in LAML (Table 1 and Figure 10B).

**TABLE 1:** Correlation between PIMERG expression and TMB, MSI.

TMB			MSI		
	
Cancer type	Cor	*p*-Value	Cancer type	Cor	*p*-Value
ACC	0.28105122	*/0.01210685	BLCA	0.13992629	**/0.00463093
BLCA	0.28028710	***/8.41E-09	BRCA	0.07904799	*/0.01111502
BRCA	0.36890862	***/9.14E-33	COAD	0.23198817	***/0.00000126
CHOL	0.37704511	*/0.02340112	HNSC	0.11359888	*/0.01134838
COAD	0.31644755	***/1.17E-10	LAML	−0.28089362	*/0.02032512
DLBC	−0.34850640	*/0.03512868	SARC	0.20612387	***/0.00097428
ESCA	−0.17804433	*/0.02429243	STAD	0.29930448	***/3.53E-09
KICH	0.42350417	***/0.00043954	TGCT	0.16929782	*/0.038349864
KIRC	0.17563993	**/0.00131239	THCA	0.11215296	*/0.012894885
LGG	0.40891257	***/1.29E-21	UCEC	0.28926152	***/7.89E-12
LUAD	0.47438087	***/1.37E-29			
LUSC	0.20681244	***/0.00000409			
MESO	0.28186320	*/0.01185028			
OV	0.20769815	***/0.00056614			
PAAD	0.41200593	***/1.47E-07			
PRAD	0.41360450	***/2.42E-21			
SARC	0.37360957	***/3.37E-09			
SKCM	0.14014035	**/0.00245533			
STAD	0.48160603	***/9.13E-23			
TGCT	0.23130290	**/0.00512273			
THCA	0.10142548	*/0.02596674			
THYM	−0.71553745	***/1.25E-19			
UCEC	0.25272071	***/4.30E-09			
UCS	0.28000892	*/0.03660465			

*Its role in tumor immunity varies by cancer type.*

These findings may contribute to the elucidation of the role of PIMREG in tumor development and serve as a reference for achieving more precise and personalized immune-based anti-tumor strategy. *P < 0.05, **P < 0.01, and ***P < 0.001.

### Correlation of PIMREG Expression With TME Across Cancers

**FIGURE 11:**
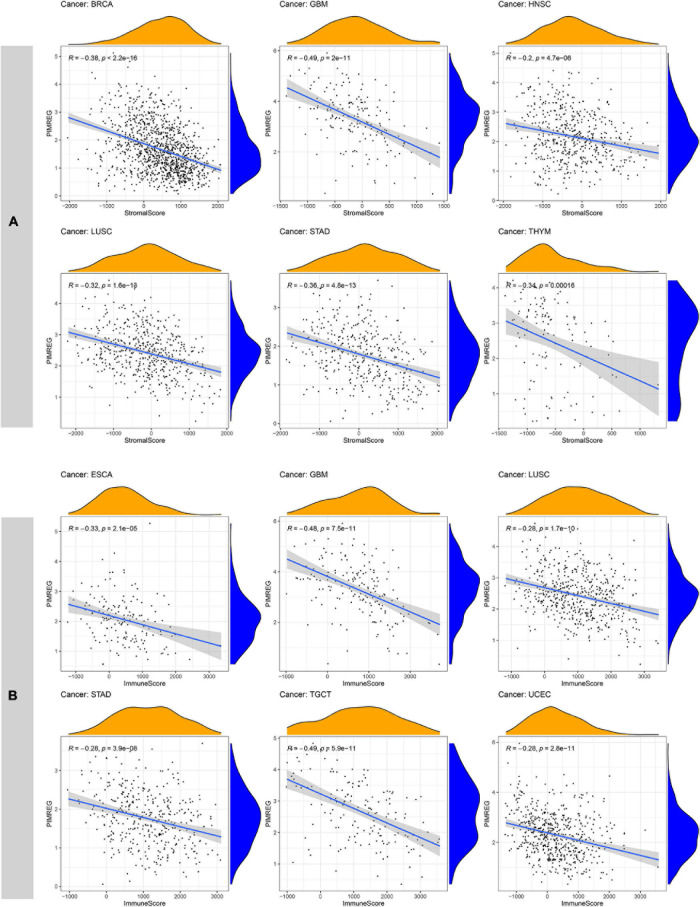
Six cancers with the highest negative correlation coefficients between PIMREG expression and TME. **(A)** Correlation between PIMREG and stromal scores in breast cancer (BRCA), glioblastoma (GBM), head and neck cancer (HNSC), lung squamous cell carcinoma (LUSC), stomach cancer (STAD) and thymoma (THYM). **(B)** Correlation of PIMREG with immune scores in esophageal cancer (ESCA), GBM, LUSC, STAD, testicular cancer (TGCT) and endometrioid cancer (UCEC).

Increasingly, reports suggest that TME plays an influential role in tumorigenesis and progression (Fane and Weeraratna, 2020). TME exerts a crucial role in the stimulation of heterogeneity across the tumor cells, thereby contributing to increased multidrug resistance and resulting in the development of cancer cell progression and metastasis (Gasser et al., 2017). Therefore, it is significant to investigate the pan-cancer associations PIMREG expression with TME. We used the ESTIMATE algorithm to evaluate stromal and immune scores for 33 cancers and analyzed the correlations of PIMREG expression levels with these two scores. The findings revealed that, in BRCA, GBM, HNSC, LUAD, LUSC, STAD, THYM, UCEC, PIMREG expression was significantly negatively correlated with stromal scores, while the expression of PIMREG positively correlated with stromal scores in KIRC and THCA (Figure 11A and Supplementary Figure 5). In addition, the expression of PIMREG was significantly, negatively related to immune scores in 11 cancer types, including ESCA, GBM, LUSC, STAD, TGCT, UCEC, CESC, LUAD, OV, PAAD, SKCM, while PIMREG was positively correlated with DLBC, KIRC, THCA (Figure 11A and Supplementary Figure 5). No significant differences were detected in other cancer types. The six cancer types with the highest correlation coefficients with a negative correlation between TME and PIMREG expression are presented in Figure 11; the results for other cancers are shown in Supplementary Table 3 and Supplementary Figure 5.

### Association of the Expression of PIMERG With the Infiltration of Immune Cells in Various Cancers

**FIGURE 12:**
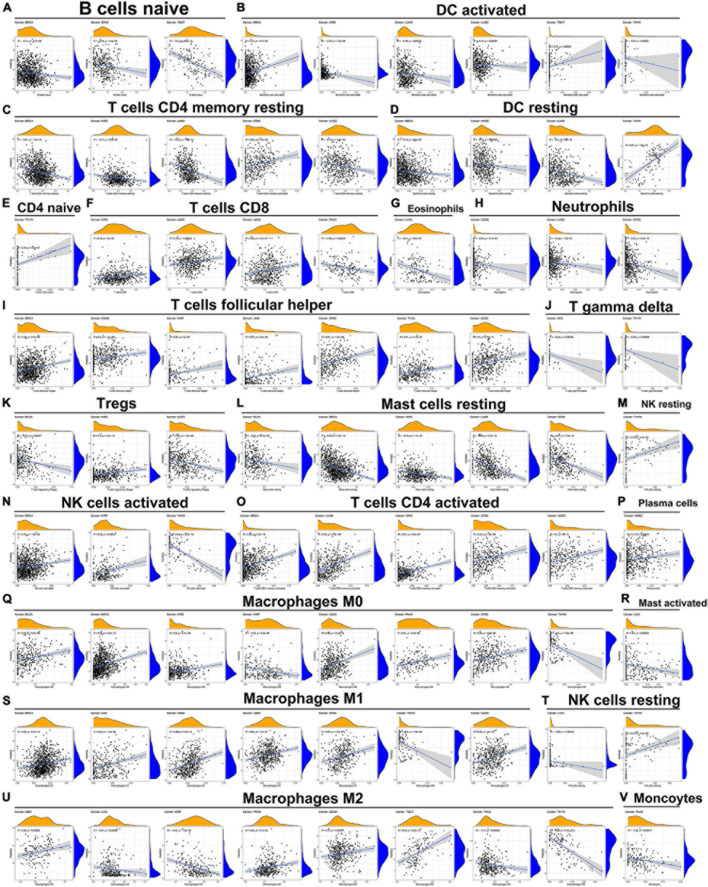
Association of PIMREG expression with infiltration levels of different immune cells in various cancers. Correlation of PIMREG with **(A)** naïve B cells, **(B)** activated DC cells, **(C)** resting memory CD4 T cells, **(D)** resting DC cells, **(E)** naïve CD4 T cells, **(F)** CD8 T cells, **(G)** eosinophils, **(H)** neutrophils, **(I)** T cells follicular helper, **(J)** gamma delta T cells, **(K)** Tregs, **(L)** resting mast cells, **(M)** resting NK cells, **(N)** activated NK cells, **(O)** activated CD4 T cells, **(P)** plasma cells, **(Q)** macrophage M0, **(R)** activated mast cells, **(S)** macrophage M1, **(T)** resting NK cells, **(U)** macrophage M2, and **(V)** monocytes in various cancers.

The relationship of PIMERG expression with the infiltrating levels of 22 immune cell subtypes was analyzed. The results showed that the infiltrating levels of immune cells correlated significantly with the expression of PIMREG in most cancer types (Supplementary Table 4). In BRCA, STAD and TGCT, the number of infiltrating naive B cells was negatively correlated with the expression of PIMREG (Figure 12A). The number of activated dendritic cells (DC) was negatively correlated with the expression of PIMREG in KIRC, LUAD, LUSC, THYM, while PIMREG expression had a positive relationship with the number of DC in BRCA and TGCT (Figure 12B). The expression of PIMREG also correlated negatively with the infiltrated levels of resting DCs in BRCA, HNSC and LUAD (except in THYM) (Figure 12D). The infiltrating levels of resting CD4 memory T cells were negatively related to the expression of PIMREG in BRCA, KIRC, LUAD, UCEC, but positively related in STAD (Figure 12C). For activated CD4 T cells, their infiltrated levels correlated negatively with the PIMREG expression in BRCA, LUAD, KIRC, STAD, and UCEC (Figure 12O). In regard to follicular helper T cells, their infiltrated levels associated positively with the expression of PIMEG in BRCA, COAD, KIRP, LIHC, STAD, THCA, and UCEC (Figure 12I). In addition, the infiltrating levels of resting mast cells had a negatively correlation with the expression of PIMREG in BLCA, BRCA, KIRC, LUAD and STAD (Figure 12L).

Further, the expression levels of PIMREG associated with several different subpopulations of invasive macrophages. For instance, the expression of PIMREG corresponded positively with the infiltration levels of M0 macrophages in BLCA, BRCA, KIRC, LUAD, PAAD, and STAD, except in KIRP and THYM (Figure 12Q). Similarly, PIMREG expression had a positive correlation with the infiltration levels of M1 macrophages in BRCA, LGG, LUAD, LUSC, STAD, and UCEC, but positively related in THYM (Figure 12S). In addition, the expression of PIMREG positively related to the infiltration of M2 macrophages in GBM, PRAD, SKCM, and TGCT, but negatively correlated in LIHC, KIRP, THCA, and THYM (Figure 12U). The correlation results of other immune cells and PIMREG expression were also presented in Figure 12.

### Co-expression of PIMREG With Immune-Related Genes and Associated Pathway Analyses in Various Cancers

**FIGURE 13:**
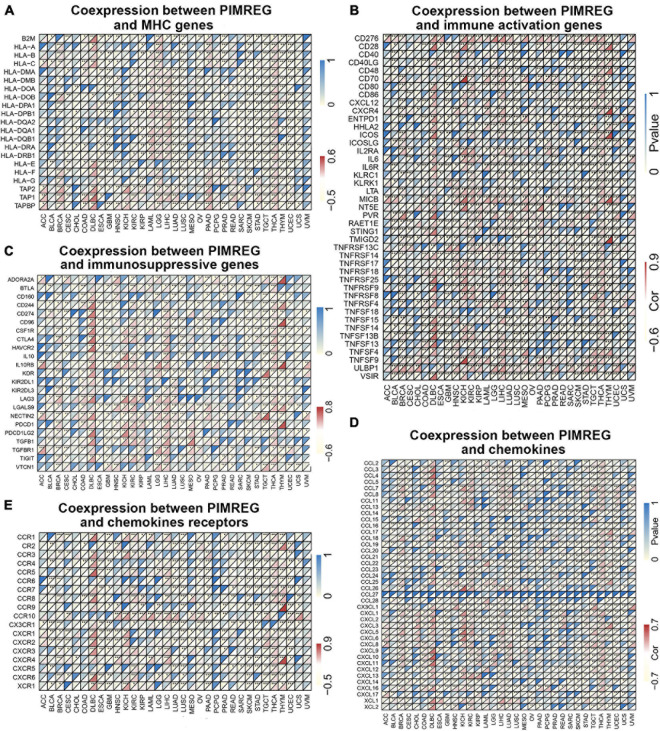
Co-expression of PIMREG with immune-associated genes. *p < 0.05, **p < 0.01, and ***p < 0.001. Coexpression between PIMREG and **(A)** MHC genes, **(B)** immune activated genes, **(C)** immunosuppressive genes, **(D)** chemokines, **(E)** chemokines receptors.

Gene co-expression analyses were further performed to investigate the correlations of PIMREG expression with immune-related genes in 33 types of cancer. The genes encoding MHC, immune activation, immune suppression, chemokine, and chemokine receptor proteins were analyzed. Heat map results showed that almost all immune-associated genes except CCL27 co-expressed with PIMREG and the major immune-related genes had a positive correlation with PIMREG in DLBC, KICH KIRC, LIHC, and THCA (Figure 13). We also found that the MHC genes had co-expression with PIMREG in almost all cancer types without READ and UVM, particularly in THCA, TGCT, UCEC, LUSC, LUAD, LIHC, LGG, and GBM (Figure 13A). In addition, immune activation genes and immunosuppressive genes were co-expressed with PIMREG in all cancer types, while the correlation in ACC, UCS, and UVM were relatively small (Figures 13B,C).

**FIGURE 14:**
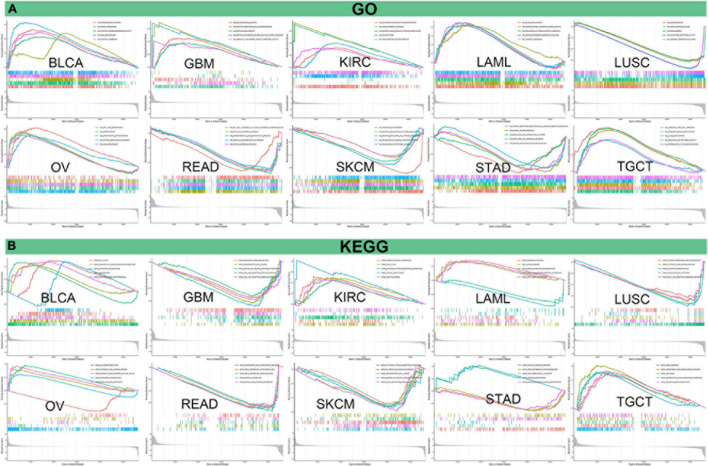
Pathway analysis of PIMREG in various cancers. **(A)** GO functional annotations of PIMREG in tumors. **(B)** KEGG pathway analyses of PIMERG in various tumors. Curves with different colors represent that PIMREG regulates distinct functions or pathways of various tumors. Peaks of curves upward indicating positive regulation and peaks of curves downward representing negative regulation.

Afterward, we analyzed GO functional annotations and KEGG pathway analyses of PIMREG in various cancers. The results of GO functional annotations and KEGG pathway analyses are shown in Figure 14 and Supplementary Figure 6. The data indicate that PIMREG negatively regulated some immune-associated functions in STAD, including immune response regulating cell surface receptor signaling, regulation of lymphocyte activation, second messenger mediated signaling (Figure 14A). In SKCM, PIMREG also negatively regulated immune-related functions, including positive regulation of cytokine production, regulation of immune effector process (Figure 14A). KEGG also demonstrated that PIMREG was able to negatively regulate several crucial immune-related pathways, such as chemokine signaling pathway, NK cell mediated cytotoxicity, NOD like receptor signaling pathway and TOLL like receptor signaling pathway in GBM; regulation of autophagy, antigen processing and presentation, and RIG-I like receptor signaling pathway in LUSC; complement and coagulation cascades in READ; cytokine cytokine-receptor interaction, FC epsilon RI signaling pathway, NK cell mediated cytotoxicity and T cell receptor signaling pathway in SKCM. Contrarily, PIMREG positively regulated cytokine cytokine-receptor interaction in KIRC; regulation of autophagy in OV; complement and coagulation cascades and TGF-β signaling pathway in TGCT (Figure 14B). In addition to immune-related pathways, PIMREG also regulates many other pathways, such as muscle system progress, VEGF pathway, drug metabolism cytochrome p450, cell cycle regulation, etc.

## Discussion

This work showed that the PIMREG gene was highly expressed in 20 cancers, and IHC results confirmed this trend at protein level. The results of CHOL, COAD, SARC, BRCA, and LUAD resembled the results of previous studies (Jiang Y. et al., 2020; Jiang Z.M. et al., 2020; Mizuno et al., 2020; Xu Z.S. et al., 2019; Yao et al., 2019). In addition, a previous study showed that PIMREG was upregulated in BLCA, BRCA, HNSC, KIRC, KIRP, LIHC, LUAD, LUSC, PRAD, THCA, and UCEC (Hu et al., 2017), which was consist with our results and was complemented by our results. Additionally, we found high expression of PIMERG in LGG, OV, SKCM, TGCT, and UCS, but insufficient expression data of normal cerebral cortex, ovary, skin, testis and uterus in TCGA. IHC analysis in the HPA indicated that PIMREG were not detected in normal cerebral cortex, ovary, testis and uterus, and it could be determined that PIMREG was highly expressed in LGG, OV, TGCT, and UCS compared to normal tissues. Hence, PIMREG is highly expressed in at least in 24 cancer types and high expression of PIMREG may be a predictive signal for tumorigenesis. Moreover, as shown in the HPA, the mRNA of PIMREG was enriched at thymus and lymphoid tissue which play crucial roles in immune system. Therefore, we speculate that PIMREG may play a role in cancer development by regulating immune system-related functions.

Kaplan–Meier survival analyses showed that high expression of PIMREG was associated with poor prognosis of BRCA. A previous research showed that promoted up-regulation of PIMREG promoted the aggressiveness of BRCA through activating NF-κB signaling (Jiang et al., 2019). In addition, overexpression of PIMREG promoted epithelial-to-mesenchymal transition and enhanced stemness features in BRCA (Zhang et al., 2019), and knockdown of PIMREG inhibited proliferation and migration of BRCA cells (Yao et al., 2019). Moreover, a clinical prospective study revealed a value of PIMREG as a biomarker and target for immunotherapy (Wang et al., 2020). Our results also showed that high expression of PIMREG was associated with a shorter survival time in CHOL patients, was consistent with a previous study that identified PIMREG as a biomarker of proliferation that facilitated aggressive development of CHOL in part by regulating cell cycle-related markers (Jiang Z.M. et al., 2020). Similarly, PAAD patients with high expression of PIMREG showed a poor prognosis which also in accordance with the findings that regarded aberrant PIMREG mRNA expression as an independent predictor of poor survival in PAAD (Jiao et al., 2019). In this study, we for the first time found that high expression of PIMREG was associated with poor prognosis in ACC, KIRC, LGG, LUAD, MESO, PRAD, and SARC. Contrastly, the high expression of PIMREG related to a better prognosis in patients with THYM, which may be related to the high PIMREG expression of the normal thymus itself.

Additionally, we found that the expression of PIMREG correlated to gender in some cancer types, including BLCA, DLBC, HNSC, KIRC, and LUAD. In these types of cancer, PIMREG was found to express generally higher in male patients compared to female, but the involved mechanisms need to be further elucidated. Afterward, we discovered that PIMREG expression correlated with age in some cancers. The expression of PIMREG was lower in older individuals with BRCA, ESCA, KIRP, LIHC, PCPG, LUAD, LUSC, and THYM, while higher PIMREG expression correlated to older patients with LUSC and STAD. These findings may have implications for the choice of immune therapy regimens for patients with different age. This work also discovered that the expression of PIMREG was associated with the stage of tumor in most tumors, and was especially different in stage I and IV cancers. In patients with ACC, BRCA, KICH, KIRC, KIRP, and LUAD, the expression of PIMREG was higher in stage IV than stage I. PIMREG expression on breast cancer cells was previously described to be in positive correlation with the pathological stage of BRCA (Jiang et al., 2019). These findings clearly indicate that PIMREG can be applied as a biomarker to identify the prognosis of a variety of cancers.

Tumor mutation burden, as a prospective pan-cancer predictive biomarker, is able to provide guidance for immune therapy in the age of precise medicine (Fumet et al., 2020). A previous study has also revealed that TMB is a pan-oncogenomic biological marker that correlates with the efficacy of ICI and that higher TMB is associated with better response to ICI and OS (Samstein et al., 2019). In addition, TMB also predicts the prognosis of patients with pan-cancer after immunotherapy (Xiang et al., 2020). MSI is also a key biological marker in ICI (Boland and Goel, 2010; Lee et al., 2019), and high-frequency MSI in COAD is a predictive factor of clinical features and prognosis (Gryfe et al., 2000). The present work indicated that the expression of PIMREG associated with TMB in 24 cancers and with MSI in 10 cancers. These results may suggest that the expression level of PIMREG affects the TMB and MSI of the tumor, thus influencing the patient’s response to the therapy of ICI. This promises to have a novel reference value for the prognosis of immune therapy in a variety of types of cancer. According to the previous works and our findings, we inferred that among tumors with positive correlation between PIMREG expression and TMB, tumors with high PIMREG expression and high TMB and MSI expression may have a better prognosis after treatment with ICI.

Tumor microenvironment characteristics can be used as markers to evaluate tumor cell responses to immune therapy and affect clinical results (Wu and Dai T., 2017). Our results showed that PIMREG plays a crucial role in cancer immunity. We performed transcriptome analysis of the TCGA database of pan-cancer data and found that PIMREG expression was significantly negatively correlated with the immune component of TME in 11 cancers, including BRCA, ESCA, GBM, LUAD, LUSC, OV, PAAD, SKCM, STAD, TGCT, and UCEC, and negatively correlated with the stromal component of TME in 6 cancers, including BRCA, GBM, HNSC, LUAD, LUSAC, STAD, THYM, and UCEC. PIMREG has been identified in a recent study as a potential target gene necessary for effective immune targeting in tumor stem cell populations of BRCA (Wang et al., 2020). Our study further elucidated that PIMREG has a wider oncologic applicability and confirms that in other cancers, the expression of PIMREG closely correlates with the biological progression of various immune cells and immune-associated cytokines. Cancer cells are known to be under the surveillance of immune cells throughout their life, and cancer develops and progresses only when the immune cells fail to destroy the preneoplastic cells (Carlsten and Järås, 2019). Hence, high expression of PIMREG in certain cancers leads to a decline in immune scores, which may lead to rapid development of cancer cells. In addition, we revealed that PIMREG is co-expressed with genes encoding MHC, immune activation, immune suppression, chemokine and chemokine receptor. All these findings suggest that the expression of PIMREG closely associated with the immune infiltration of tumor cells, affecting the prognosis and providing a new target for the improvement of immunotherapy for various types of cancer patients.

At present, there are very few studies on the immunological role of PIMREG in cancer and PIMREG is commonly thought to be a cell cycle promoter in hypoxic fetal cardiac myocytes (Hashimoto et al., 2017). PIMREG was also reported to promote the aggressiveness of BRCA through disrupting the NF-κB/IκBα negative feedback loop (Jiang et al., 2019). It is worth noting that the activation of NF-κB has been proved to be involved in the macrophage polarization and inflammatory cytokines (Ye et al., 2019; Zhu et al., 2021). In addition, PIMREG is known to regulate Th17 differentiation and inflammation-associated cancer by activating STAT3 (Xu Z.S. et al., 2019). Activation of STAT3 leads to the production of downstream pro- and anti-inflammatory cytokines that play an important role in the pathological development of tumors (Owen et al., 2019). Our enrichment analysis for the first time demonstrated that PIMREG can potentially influence the etiology or pathogenesis of cancer by acting on immune-related pathways; chemokine pathway, NK cell mediated cytotoxicity, NOD like receptor signaling pathway, TOLL like receptor signaling pathway, antigen processing and presentation, regulation of autophagy, RIG-I like receptor signaling pathway; complement and coagulation cascades, cytokine cytokine-receptor interaction, FC epsilon RI signaling pathway, NK cell mediated cytotoxicity and T cell receptor signaling pathway, etc.

In conclusion, our first pan-cancer analysis of PIMREG shows the presence of high expression of this gene in most tumor tissues compared to normal tissues and revealed a correlation of PIMREG expression with clinical prognosis. The results suggest that PIMREG may act as an independent prognostic factor in a number of cancers, and that its high expression levels in major tumors are associated with poor prognostic outcomes, but further investigation of the specific role of PIMREG in each tumor is still needed. In addition, PIMREG expression is correlated with TMB, MSI and the infiltration of immune cells in various cancers. Its role in tumor immunity varies by cancer type. These findings may contribute to the elucidation of the role of PIMREG in tumor development and serve as a reference for achieving more precise and personalized immune-based anti-tumor strategy.

## Data Availability Statement

The datasets presented in this study can be found in online repositories. The names of the repository/repositories and accession number(s) can be found in the article/Supplementary Material.

## Author Contributions

XX and LG have designed the study. HZ and XH have written the article. YY, YZ, and ZJ have analyzed the data. All authors have read and approved it for publication.

## Conflict of Interest

The authors declare that the research was conducted in the absence of any commercial or financial relationships that could be construed as a potential conflict of interest.

## Publisher’s Note

All claims expressed in this article are solely those of the authors and do not necessarily represent those of their affiliated organizations, or those of the publisher, the editors and the reviewers. Any product that may be evaluated in this article, or claim that may be made by its manufacturer, is not guaranteed or endorsed by the publisher.

## Footnotes

1
https://www.oncomine.org/

2 https://xena.ucsc.edu/, derived from the TCGA

3
https://tcga.xenahubs.net

4
https://portals.broadinstitute.org/ccle

5
http://www.proteinatlas.org/

6
https://www.gsea-msigdb.org/gsea/downloads.jsp

## Supplementary Material

Supplementary Figure 1 Association of PIMREG expression with gender in various cancers.

Supplementary Figure 2 Association of PIMREG expression with age in various cancers.

Supplementary Figure 3 Association of PIMREG expression with age in various cancers.

Supplementary Figure 4 Association between the expression of PIMREG and tumor stage in various cancers.

Supplementary Figure 5 Correlation between PIMREG and stromal scores in various cancer types.

Supplementary Figure 6 KEGG and GO pathway analysis of PIMREG in various cancers.
