# Supplementary figures and images for: Pan-Cancer Analysis of PIMREG as a Biomarker for the Prognostic and Immunological Role
Source: Front Genet. 2021 Sep 14;12:687778. doi: 10.3389/fgene.2021.687778 (PMC8477005; doi:10.3389/fgene.2021.687778)

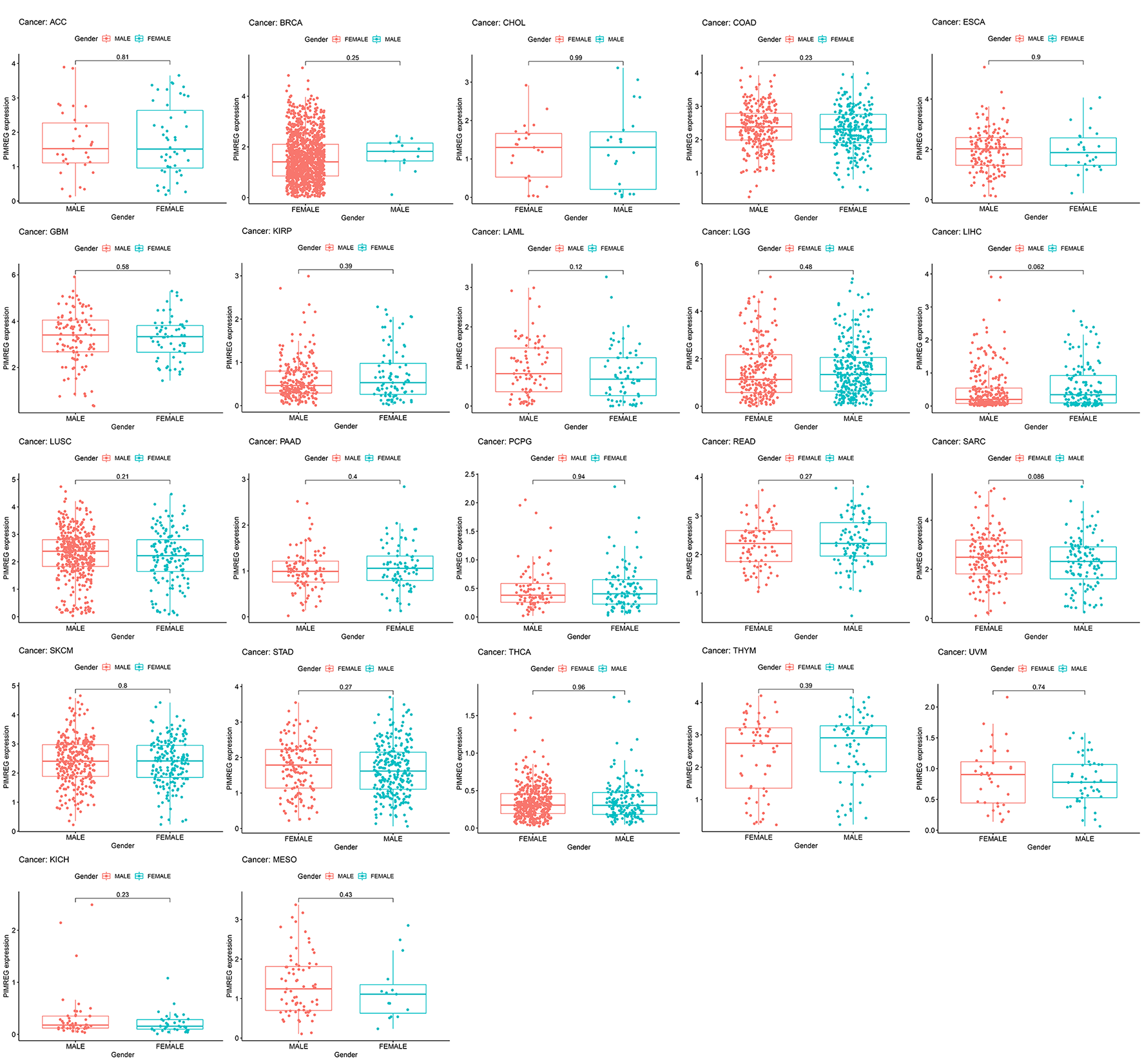

Supplement: Supplementary Figure 1 — Association of PIMREG expression with gender in various cancers. [file Image_1.TIF]

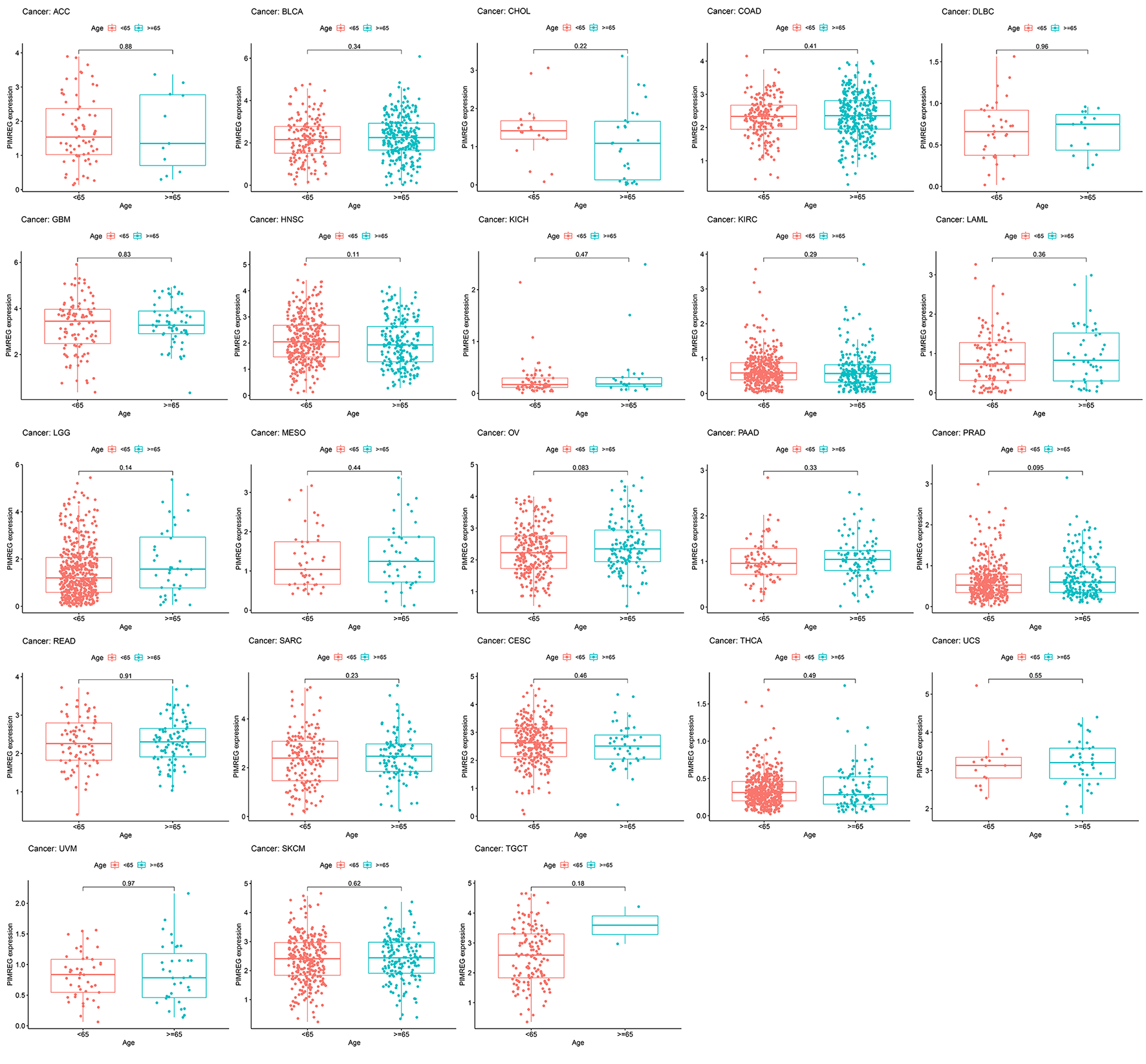

Supplement: Supplementary Figure 2 — Association of PIMREG expression with age in various cancers. [file Image_2.TIF]

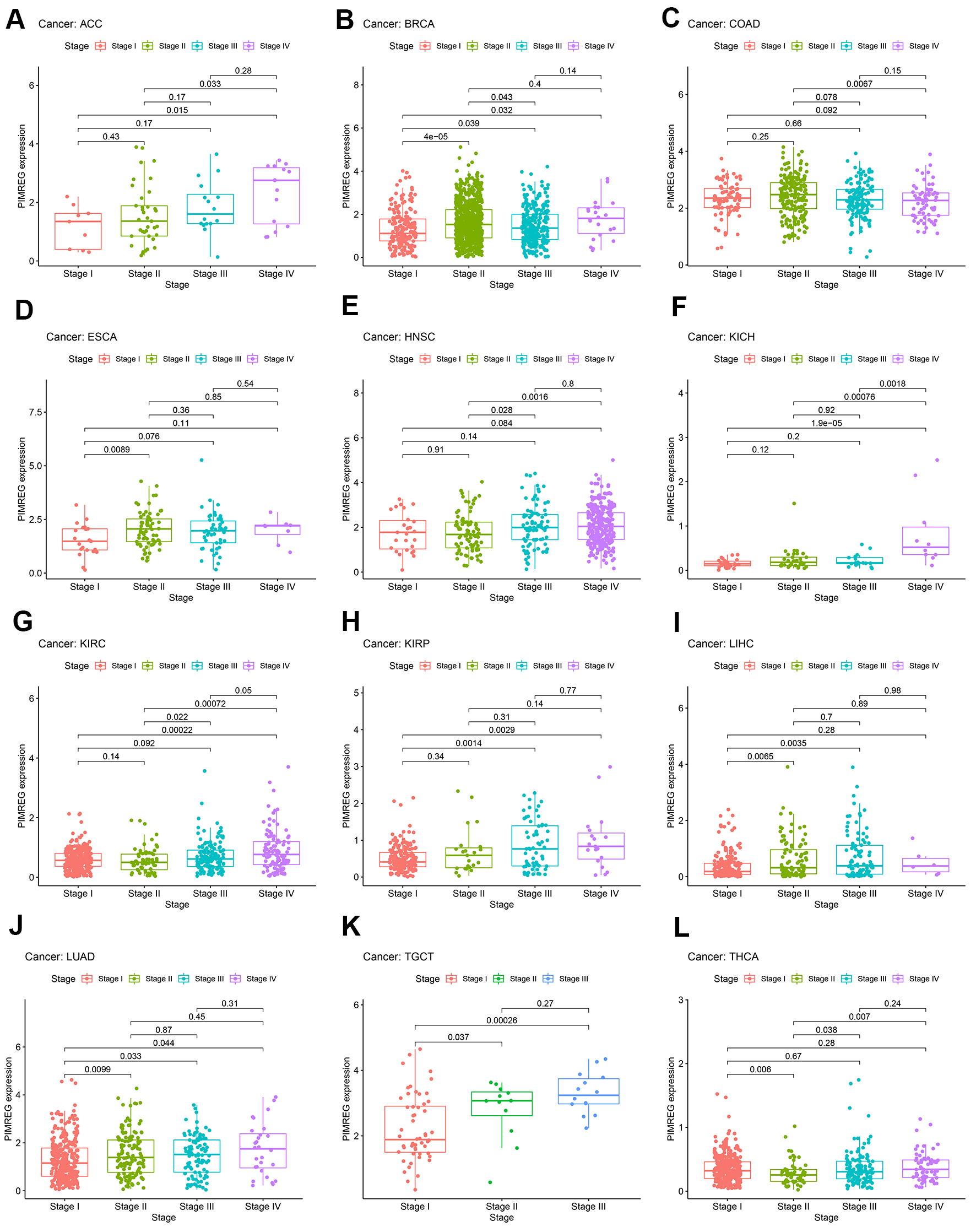

Supplement: Supplementary Figure 3 — Association of PIMREG expression with age in various cancers. [file Image_3.TIF]

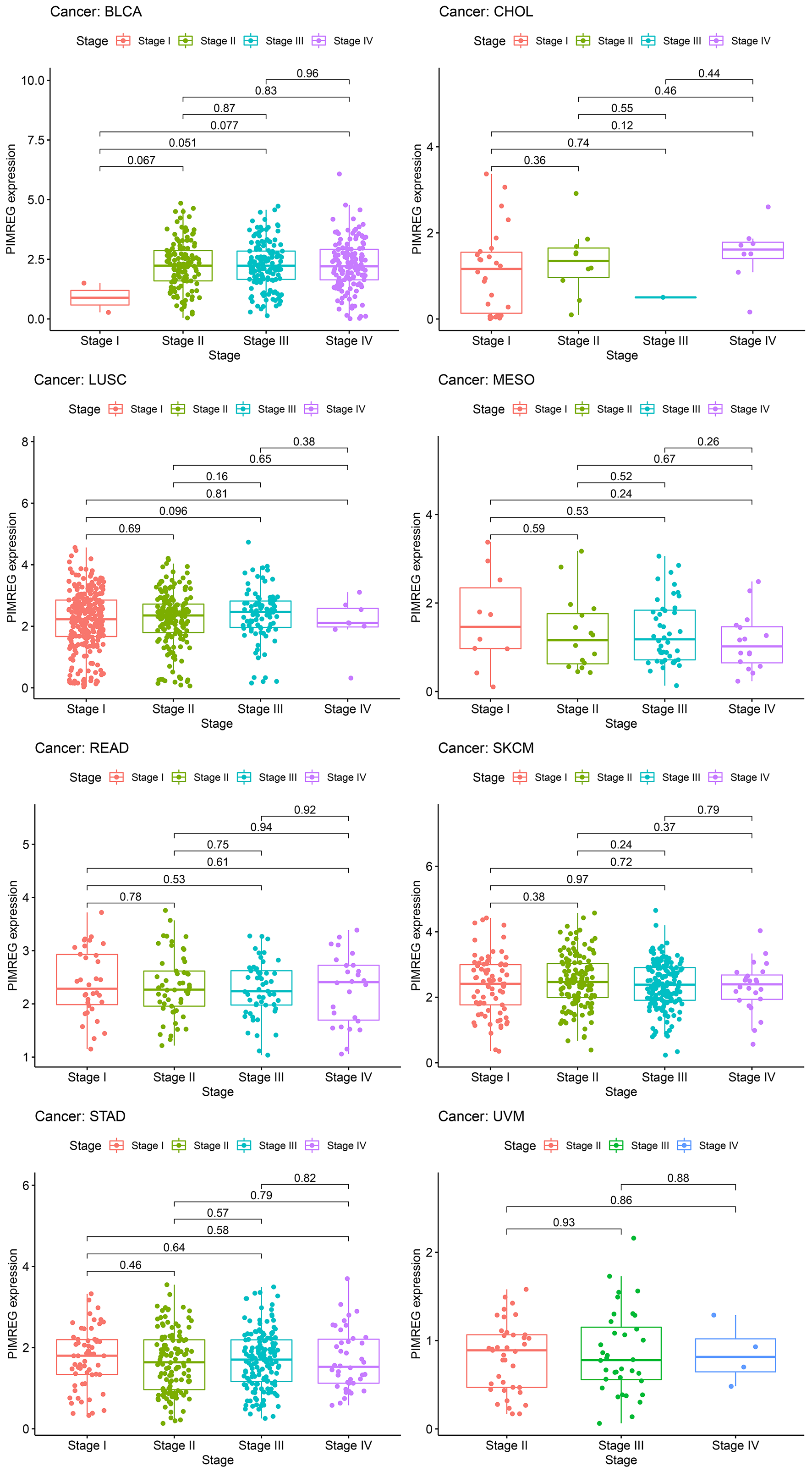

Supplement: Supplementary Figure 4 — Association between the expression of PIMREG and tumor stage in various cancers. [file Image_4.TIF]

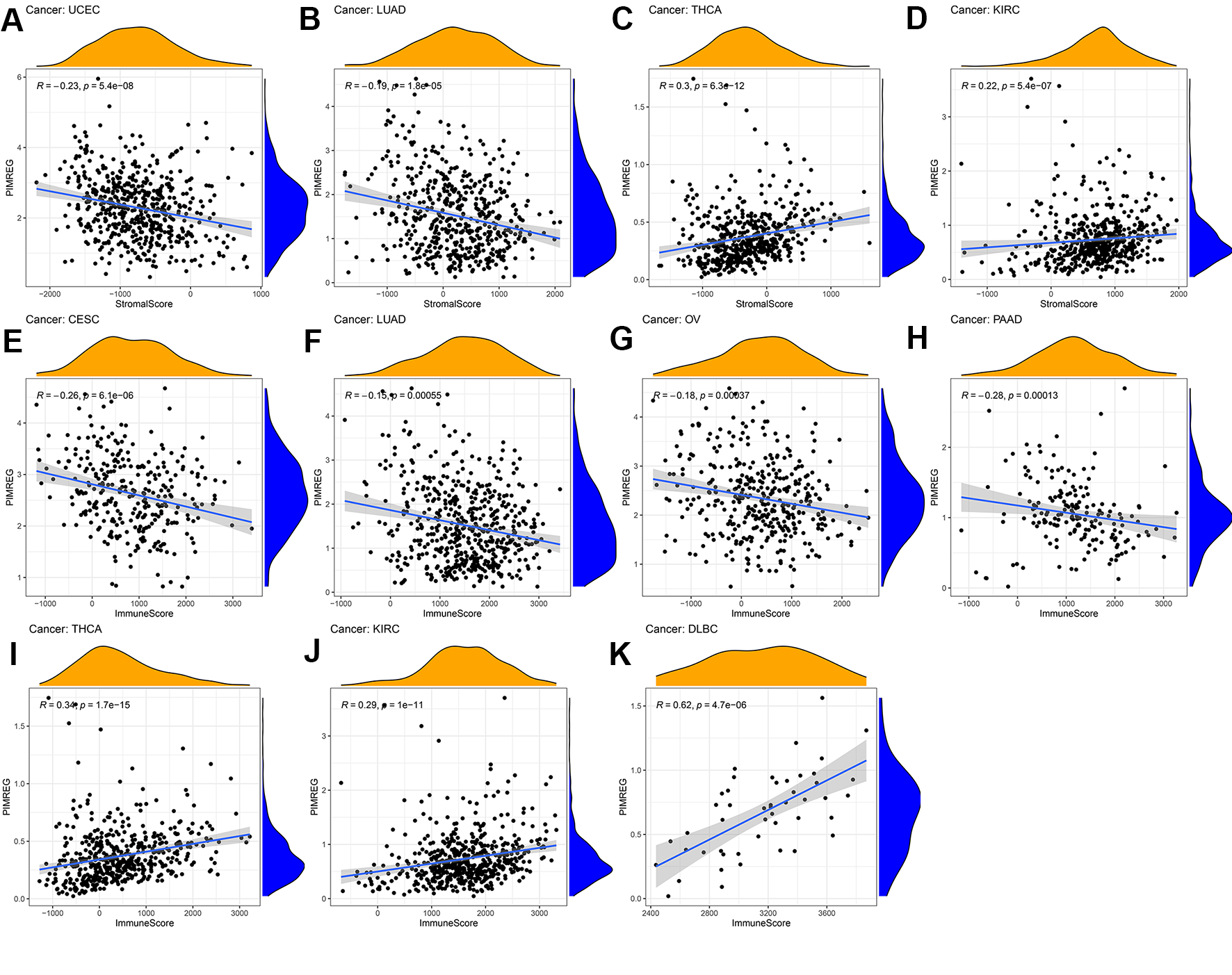

Supplement: Supplementary Figure 5 — Correlation between PIMREG and stromal scores in various cancer types. [file Image_5.TIF]

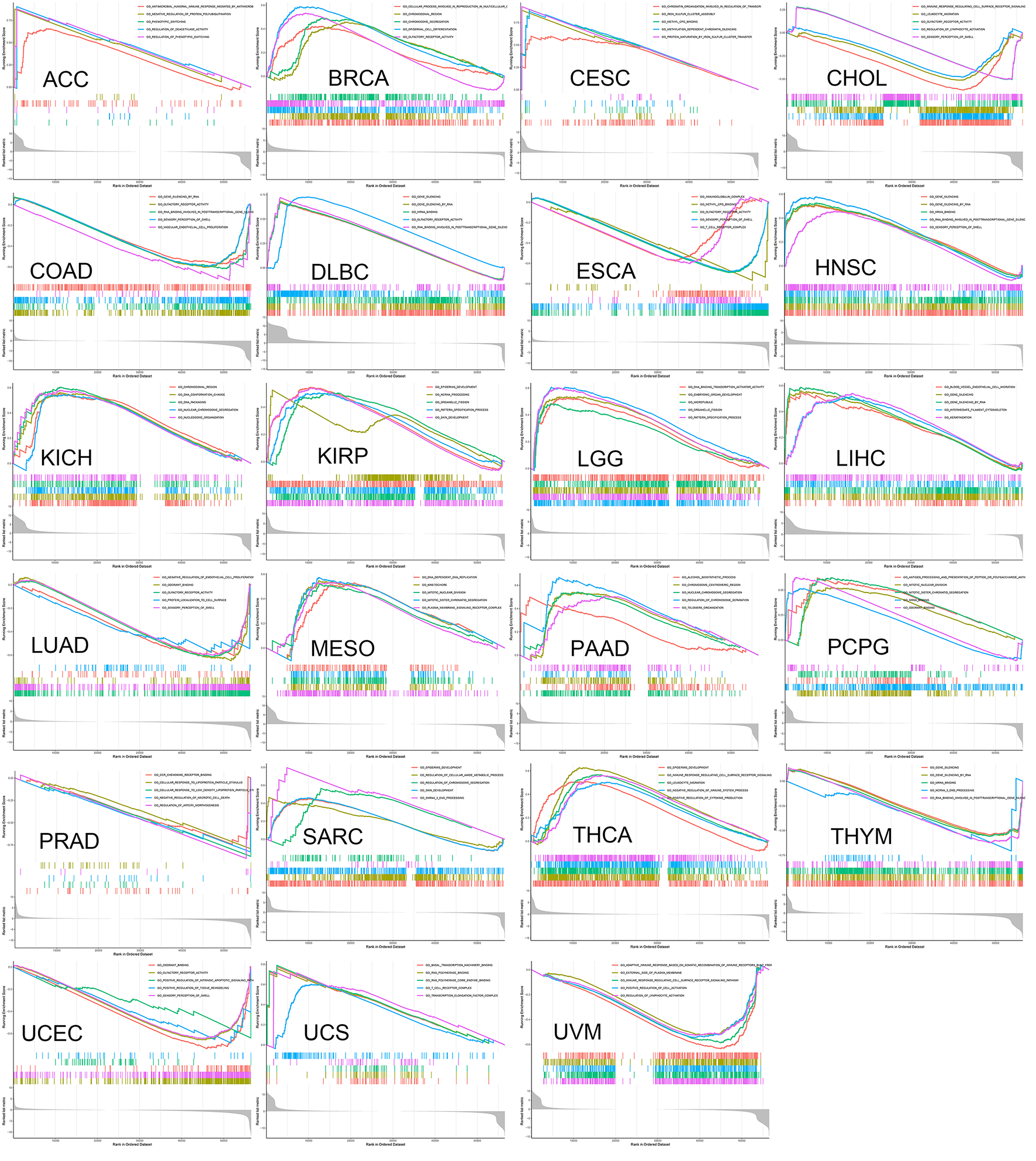

Supplement: Supplementary Figure 6 — KEGG and GO pathway analysis of PIMREG in various cancers. [file Image_6.TIF]
