# Supplementary material for: Pan-Cancer Analysis of PIMREG as a Biomarker for the Prognostic and Immunological Role
Source: Front Genet. 2021 Sep 14;12:687778. doi: 10.3389/fgene.2021.687778 (PMC8477005; doi:10.3389/fgene.2021.687778)
Supplement: Supplementary file 7 [file Data_Sheet_1.PDF]

Table S1. The detail information of 33 cancers.

Cancer type	Number of normal samples	Number of cancer samples
ACC	0	79
BLCA	19	411
BRCA	113	1104
CESC	3	306
CHOL	9	36
COAD	41	471
DLBC	0	48
ESCA	11	162
GBM	5	168
HNSC	44	502
KICH	24	65
KIRC	72	535
KIRP	32	289
LAML	0	151
LGG	0	529
LIHC	50	374
LUAD	59	526
LUSC	49	501
MESO	0	86
OV	0	379
PAAD	4	178
PCPG	3	183
PRAD	52	499
READ	10	167
SARC	2	263
SKCM	1	471
STAD	32	375
TGCT	0	156
THCA	58	510
THYM	2	119
UCEC	35	548
UCS	0	56
UVM	0	80
