# Supplementary material for: Pan-Cancer Analysis of PIMREG as a Biomarker for the Prognostic and Immunological Role
Source: Front Genet. 2021 Sep 14;12:687778. doi: 10.3389/fgene.2021.687778 (PMC8477005; doi:10.3389/fgene.2021.687778)
Supplement: Supplementary file 9 [file Data_Sheet_3.PDF]

Table S3. Association between PIMREG expression and and tumor immune microenvironment in pan-cancer.

Cancer type	Gene	StromalScore	ImmuneScore
ACC	PIMREG	0.063826062	0.311491814
BLCA	PIMREG	0.013569699	0.002479455
BRCA	PIMREG	0	0.6089586
CESC	PIMREG	0.006842844	6.14E-06
CHOL	PIMREG	0.021587221	0.037706166
COAD	PIMREG	0.001738815	0.468416781
DLBC	PIMREG	0.006032626	4.70E-06
ESCA	PIMREG	0.239454992	2.12E-05
GBM	PIMREG	2.02E-11	7.51E-11
HNSC	PIMREG	4.65E-06	0.00457297
KICH	PIMREG	0.433404647	0.104081894
KIRC	PIMREG	5.36E-07	1.04E-11
KIRP	PIMREG	0.238652057	0.031873085
LAML	PIMREG	0.017360548	0.208616475
LGG	PIMREG	0.267241701	0.109620422
LIHC	PIMREG	0.607872957	0.001355649
LUAD	PIMREG	1.79E-05	0.0005538
LUSC	PIMREG	1.60E-13	1.72E-10
MESO	PIMREG	0.035666355	0.473339307
OV	PIMREG	0.007178643	0.000367384
PAAD	PIMREG	0.001067924	0.00013091
PCPG	PIMREG	0.126856528	0.77802999
PRAD	PIMREG	0.300811717	0.326533367
READ	PIMREG	0.003652002	0.518831549
SARC	PIMREG	0.004830019	0.016281235
SKCM	PIMREG	0.019371748	0.000112755
STAD	PIMREG	4.79E-13	3.88E-08
TGCT	PIMREG	0.0700452	5.91E-11
THCA	PIMREG	6.26E-12	1.72E-15
THYM	PIMREG	0.000163921	0.002784062
UCEC	PIMREG	5.36E-08	2.83E-11
UCS	PIMREG	0.562026054	0.068900999
UVM	PIMREG	0.278763436	0.357813521
